# Beyond job satisfaction: a job embeddedness-based mediation model to explain turnover intention in Chinese social workers

**DOI:** 10.3389/fpsyg.2026.1766949

**Published:** 2026-02-20

**Authors:** Yang Luo, Xiaoge Zhao

**Affiliations:** 1 Department of Social Work and Social Policy, School of Sociology, Nankai University, Tianjin, China; 2 HNU-ASU International College, Hainan University, Haikou, Hainan, China

**Keywords:** Job Demands-Resources model, job embeddedness, organizational working conditions, Price-Mueller model, social worker, turnover intention

## Abstract

**Background:**

The Price-Mueller model and the Job Demands-Resources model examine how organizational working conditions influence turnover intention through the mediating mechanisms of subjective experiences, such as job satisfaction and burnout.

**Methods:**

To address the specific needs of indigenization and professionalization, we constructed a model incorporating “job embeddedness” based on existing models to better predict Chinese social works' turnover intention. This study used Partial Least Squares Structural Equation Modeling (PLS-SEM) to test the proposed model.

**Result:**

The model showed good fit indices. organizational working conditions significantly influenced turnover intention. The mediating effects of job embeddedness and job satisfaction were significant across most pathways. Job embeddedness demonsrated the strongest explanatory power for turnover intention within the mode.

**Conclusions:**

Strategies for reducing social workers' turnover intention should not only consider the job satisfaction pathway but, more importantly, prioritize the stronger mediating mechanism of job embeddedness linking organizational working conditions to turnover.

## 1 Introduction

Social work is a fundamental component of the social welfare system and a provider of welfare delivery (Payne, 2006). However, the social work sector in China currently faces a severe challenge regarding talent retention. Data from the 2019 China Social Work Longitudinal Study (CSWLS 2019) reveals that 28.8% of surveyed social workers have considered quitting, while 35.4% remain undecided; only 35.8% have no intention of leaving. Moreover, approximately 18% of social workers plan to leave the industry entirely within the next 3 years.

Turnover intention is widely recognized as the cognitive antecedent of actual turnover behavior (Michaels and Spector, 1982; Lee and Mowday, 1987), with empirical studies typically demonstrating a correlation of approximately 0.50 between turnover intention and actual turnover behavior (Griffeth et al., 2000; Steel and Ovalle, 1984). Given the methodological difficulties in tracking actual turnover behavior, this study uses turnover intention as the dependent construct.

The Price-Mueller (PM) and Job Demands-Resources (JD-R) models are well-established frameworks for understanding turnover intention. Regarding exogenous factors, both models identify organizational conditions as critical drivers; as to endogenous factors, both models posit job satisfaction as a core mediator, while focusing individually on organizational commitment (PM) and job burnout (JD-R). However, relying solely on such endogenous variables fails to capture the complexity of turnover decisions and offers limited explanatory power for an employee's decision to stay or leave (Locke, 1976; Porter and Steers, 1973).

To address the limitation, (Mitchell et al. 2001) introduced the concept of “job embeddedness,” a relatively exogenous construct. This concept describes an employee's position within a web of work and social networks, reflecting the structural forces and costs that an individual faces when attempting to “detach” from an organization. Existing research indicates that “job embeddedness” explains incremental variance in turnover intention beyond traditional attitudinal predictors, such as “job satisfaction” and “affective commitment” (Mitchell et al., 2001; Holtom and Inderrieden, 2006; Jiang et al., 2012).

China is a “*Guanxi* society”. Chinese sociologists note that the concept of *Guanxi* holds far greater social significance and complexity in the Chinese context than similar concepts in the West (Zhou, 2018). In the workplace, the operation of Guanxi is viewed as an informal but highly effective organizational strategy (DiTomaso and Bian, 2018), and social work has centered on the power of Guanxi.

Based on the specific characteristics of the social work profession, this study integrates the PM and JD-R models and proposes a dual-mediation model that synthesizes endogenous subjective emotional experiences and relatively exogenous cognitive appraisals of the network. This approach aims to overcome the limitations of models that rely purely on attitudinal perspectives, thereby providing a more comprehensive theoretical explanation and empirical basis for understanding social worker turnover.

## 2 Literature review and hypotheses

### 2.1 Job embeddedness and social workers' turnover intention

Job embeddedness, which is conceived as a key mediating construct between on-the-job factors and turnover, refers to the extent to which an employee is enmeshed in a web of work and social networks. It consists of three key components: “Links,” “Fit,” and “Sacrifice” (Mitchell et al., 2001). Organizational embeddedness is defined as “on-the-job” forces, while community embeddedness is defined as “off-the-job” forces (Mitchell et al., 2001). “Links” refer to the extent of an individual's connections to other people or activities; “Fit” denotes the degree to which the work environment and community align with or suit the individual's life space; and “Sacrifice” represents the perceived cost of leaving the job (Mitchell et al., 2001).

Job embeddedness extends beyond traditional affective approaches and offers unique applicability for examining social workers' turnover. Social work views the premise that “human beings are relational beings” as the theoretical origin (Qin and Xu, 2025) and attempts to intervene in social networks where the client serves as the focal point. Within the field of practice, positive interaction loops inevitably turn social workers and other actors into nodes within each other's social networks. As professional and private networks expand and intertwine, habitus emerges within the field. Habitus refers to a system of durable, transposable dispositions—including values, cognitive structures, and emotional tastes—formed through historical social relations (Lizardo, 2004). Leaving a job implies severing the network deeply bound to this habitus (Liu and He, 2023), which undoubtedly constitutes a significant “Sacrifice” for social workers. The construct of job embeddedness systematically describes these structural forces through the dimensions of “Links,” “Fit,” and “Sacrifice”. Therefore, this study posits that job embeddedness is highly significant for understanding social worker turnover.

Job embeddedness includes both organizational and community dimensions. In this study, we focus on organizational embeddedness, which refers to the “on-the-job” forces within a specific organization that retain employees. It is distinct from community embeddedness, which refers to “off-the-job” forces binding a person to their physical environment. Since this study examines the impact of workplace conditions, organizational embeddedness is selected as the mediating construct because it occurs within the work domain and is significantly more controllable by the organization than community-based factors.

### 2.2 The relationship between working conditions and turnover intention

Both the PM and JD-R models examine the relationship between working conditions and turnover intention. The PM model categorizes determinants of turnover intention into external environmental factors, individual factors, and organizational environmental factors. The latter includes salary, job support, job stress, and job autonomy. The JD-R model classifies job characteristics into job demands and job resources. Job demands refer to the physical and psychological effort required by the job (e.g., job stress, emotional burden). In contrast, job resources refer to the physical, psychological, and organizational assets used to achieve work goals (e.g., work support, job significance). Based on these models and data availability in the CSWLS 2019, we selected salary, job stress, and Work support as proxy variables for “organizational working conditions.”

From the perspective of the “rational economic man” hypothesis, individuals seek to maximize their economic self-interest; thus, salary is a decisive factor in retention. Numerous studies have demonstrated that salary is negatively correlated with social workers' turnover intention (Liu and He, 2023; Tang and Fei, 2020; Luo and Chui, 2020).

Conservation of Resources (COR) theory posits that work support serves as a reservoir from which individuals can acquire external resources and store coping energy (Hobfoll et al., 1990). As part of the workplace work support system, organizational support satisfies employees' emotional needs and enhances organizational identification and belonging, thereby reducing turnover intention. Empirical studies widely support a negative association between work support and turnover intention (Tang and Fei, 2020; Hu and Shi, 2022).

Job stress is another critical factor. (Demerouti et al. 2001) argue that stress arises when an individual's resources are insufficient to meet job demands. Once stress exceeds a personal threshold, individuals trigger defensive mechanisms that manifest in irrational or aggressive behaviors, or even turnover (Hobfoll, 1989). Existing research extensively confirms that job stress has a significant positive impact on social workers' turnover intention (Li et al., 2025; Wooten et al., 2011).

Accordingly, we propose the following hypotheses.

Hypothesis 1 (H1a). Job stress is positively related to turnover intention.

Hypothesis 2 (H1b). Work support is negatively related to turnover intention.

Hypothesis 3 (H1c). Salary level is negatively related to turnover intention.

### 2.3 The mediating role of job satisfaction

The PM model posits that organizational working conditions—such as salary, support, and stress—influence turnover intention via job satisfaction (Price, 2001). Job satisfaction, defined as the degree to which employees like their work (Agho et al., 1993), has been consistently shown to be negatively correlated with turnover (Tett and Meyer, 1993; Mobley, 1977; Arnold and Feldman, 1982; Bluedorn, 1982; Hollenbeck and Williams, 1986).

First, studies indicate that job stress (stemming from resource scarcity, role ambiguity, role conflict, and work overload) negatively affects job satisfaction (Price, 1977; Hakim and Sudarmiatin, 2018). Second, ample research demonstrates that employees with higher support tend to complete tasks more efficiently and effectively, boosting satisfaction and reducing turnover intention (Mor Barak et al., 2001; Choi et al., 2021). Third, salary is often a primary concern for employees. Some scholars have verified that job satisfaction mediates the relationship between salary and turnover intention (Saeed et al., 2023; Hanifa et al., 2024). Accordingly, we propose the following hypotheses.

Hypothesis 4 (H2a). Job satisfaction mediates the relationship between job stress and turnover intention.

Hypothesis 5 (H2b). Job satisfaction mediates the relationship between work support and turnover intention.

Hypothesis 6 (H2c). Job satisfaction mediates the relationship between salary level and turnover intention.

### 2.4 The mediating role of job embeddedness

Compared to the well-established “working conditions—job satisfaction—turnover intention” path, the mechanism by which working conditions influence turnover intention via job embeddedness has not yet been integrated into a holistic theoretical model, only fragmented studies have explored relationships between specific working conditions, embeddedness, and turnover.

Regarding job stress, (Kang 2022) surveyed 223 rural nurses in South Korea and found that job embeddedness mediated the positive effect of job stress on turnover intention. (Zhu and Zhang 2016) found similar results in a study of 302 employees in Anhui, China.

Regarding work support, (Karatepe 2013) used SEM on data from 174 hotel employees in Iran, confirming that job embeddedness fully mediated the effect of workplace support on turnover intention. (Harris et al. 2011) reached the same conclusion by analyzing data from 205 auto dealership workers in the US, finding that leader-member exchange (LMX) indirectly influenced turnover intention via job embeddedness.

Regarding salary, (Bambacas and Kulik 2013) analyzed data from 308 managers in a Chinese steel company and found that salary growth increased embeddedness, thereby reducing turnover intention. (Bergiel et al. 2009) also noted that job embeddedness mediates the relationship between compensation and turnover intention. Accordingly, we propose the following hypotheses.

Hypothesis 7 (H3a). Job embeddedness mediates the relationship between job stress and turnover intention.

Hypothesis 8(H3b). Job embeddedness mediates the relationship between work support and turnover intention.

Hypothesis 9 (H3c). Job embeddedness mediates the relationship between salary and turnover intention.

### 2.5 The relationship between job embeddedness and job satisfaction

Job embeddedness can be viewed as a “cognitive representation” of an individual's structural position within a social network, reflecting their subjective perception of the breadth and depth of their connection to the organization. In contrast, job satisfaction focuses more on the subjective affective evaluation of job content and environment.

Psychological theory categorizes mental processes into three stages: cognition, affect, and volition (Guo and Chen, 2008). The cognitive process is the internal mechanism by which the brain receives and processes external information; the affective process refers to the subjective experiences that accompany information processing; and the volitional process is the internal drive propelling one toward goals, manifested in external behavior (Chen, 1983).

From the perspective of the temporal and logical sequence of cognition and affect, after employees enter an organization, they form a preliminary cognition of “job embeddedness” through contact with the environment, interpersonal networks, and institutional structures. Specifically, they ask: What is the state of the work network? Do organizational values match? What would be the cost of leaving? This cognitive framework subsequently influences their overall affective response and attitudinal orientation toward the job.

(Ramaite et al. 2022), analyzing data from 213 paper mill employees in South Africa, and (Yu et al. 2020), analyzing 324 hotel employees in South Korea, both point out that job embeddedness positively influences job satisfaction. Therefore, this study infers that job embeddedness likely has a significant positive impact on job satisfaction. Accordingly, we propose the following hypotheses.

Hypothesis 10 (H4a). Job embeddedness and job satisfaction play a serial mediating role between job stress and turnover intention.

Hypothesis 11 (H4b). Job embeddedness and job satisfaction play a serial mediating role between work support and turnover intention.

Hypothesis 12 (H4c). Job embeddedness and job satisfaction play a serial mediating role between salary level and turnover intention.

Based on the literature review above, this study constructs an integrated theoretical model by synthesizing the PM and JD-R models with Job Embeddedness theory to examine the factors influencing Chinese social workers' turnover intention.

First, this study tries to improve traditional turnover models that rely solely on endogenous attitudinal variables by introducing job embeddedness as a critical mediator. Second, unlike fragmented studies that treat embeddedness and satisfaction in isolation, this model posits a serial mediation path. It suggests that working conditions first shape an employee's cognitive assessment of their structural position (embeddedness), which subsequently influences their affective evaluation, ultimately determining their intention to stay or leave. Third, this model is specifically contextualized within the landscape of Chinese social work. Recognizing the cultural significance of *Guanxi* and the relational nature of the social work profession, the inclusion of job embeddedness provides a theoretical lens that aligns with the specific habitus of Chinese social workers.

**Figure 1:**
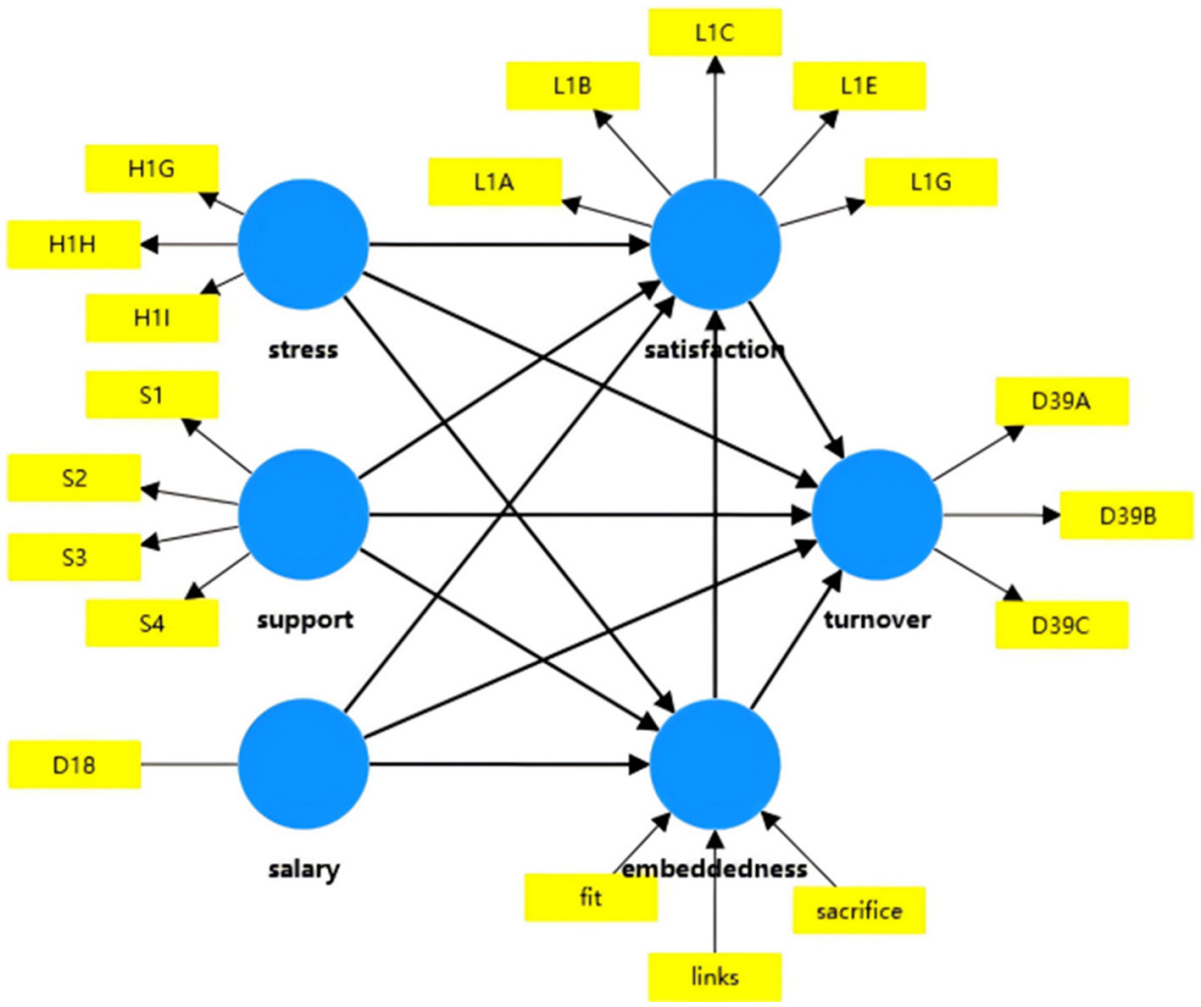
The proposed hypothetical model of this study.

Figure 1 illustrates the proposed hypothetical model of this study.

## 3 Data and methods

### 3.1 Sample selection and data source

The data used in this study are from the China Social Worker Longitudinal Study (CSWLS) database. This large-scale, continuous sampling and research project, initiated by East China University of Science and Technology, is the first of its kind in China to focus on the development dynamics of the social work profession. As of the submission date of this paper, CSWLS 2019, which this study utilizes, is the most recent wave of data available. CSWLS 2019 conducted its inaugural questionnaire survey between June and October 2019 across 56 cities nationwide in China. A multi-stage random sampling method was employed, using the registered social work organizations in each sampled city as the sampling frame. Organizations were randomly selected, and social workers were then randomly sampled within them.

**Figure 2:**
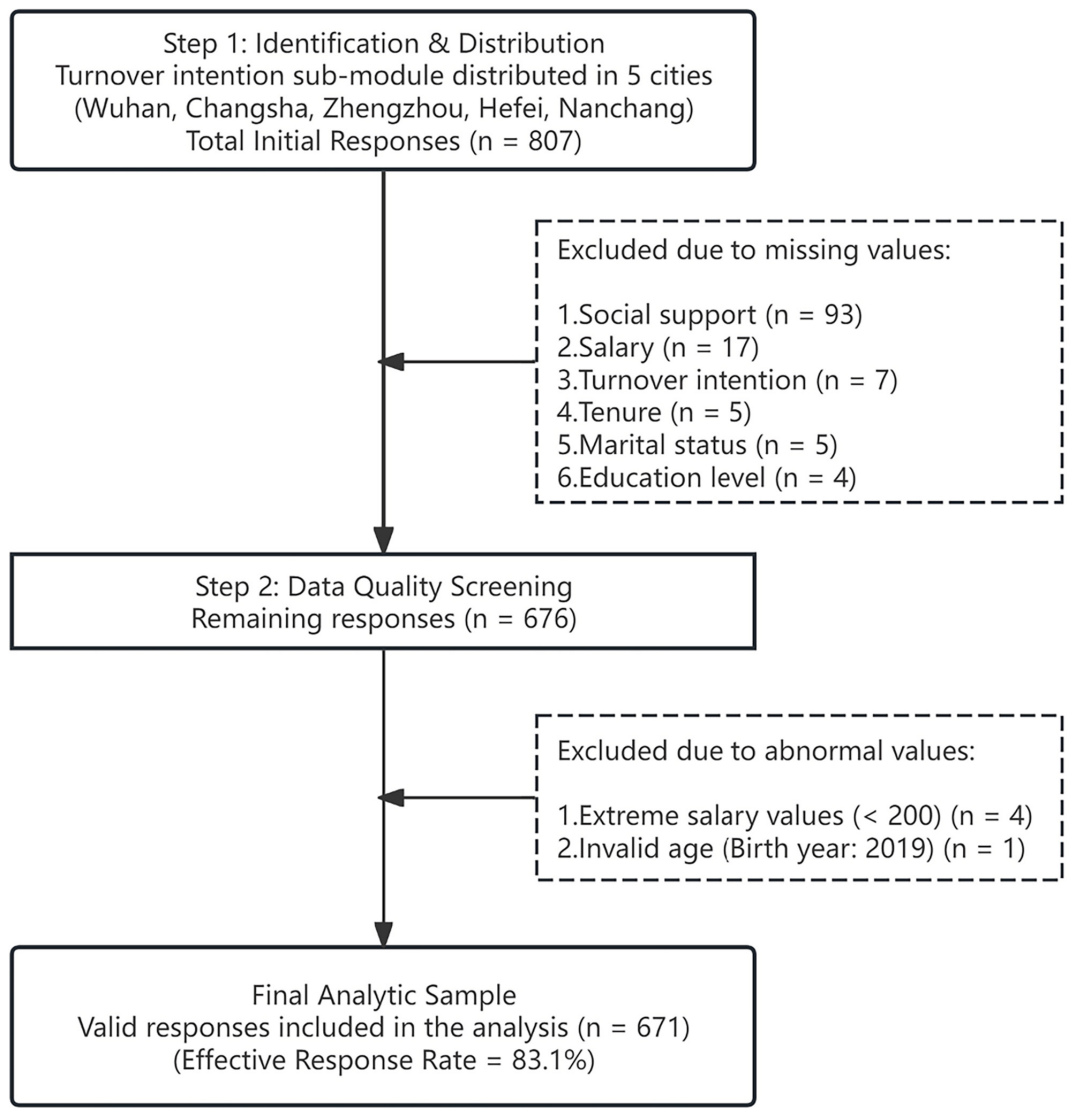
Flowchart of the sample selection process.

The turnover intention sub-module was only distributed to five cities in China (Wuhan, Changsha, Zhengzhou, Hefei, and Nanchang), yielding a total of 807 initial responses. To ensure data quality, we rigorously screened the data and excluded samples that were invalid. This process resulted in a final analytic sample of 671 valid responses. The explicit sample selection process is depicted in Figure 2.

### 3.2 Demographic characteristics of the samples

Regarding demographic characteristics, the sample was predominantly female, with males accounting for 21.5%. In terms of marital status, 52.9% of respondents were unmarried, while 47.1% were married. The participants represented a relatively young cohort, with ages ranging from 18 to 64 years; notably, the majority fell within the 18–30 age group.

Regarding educational attainment, more than half of the participants held a bachelor's degree. Those with a junior college education (or an associate degree) accounted for 25.9%, followed by those with a postgraduate degree or higher. Only a small minority had a high school education, a middle school education, or lower.

Regarding organizational tenure, the vast majority of respondents had worked in their current organization for 5 years or less. A further 6.0% reported a tenure of 6–10 years, while only 3.7% had worked for more than 10 years. This distribution suggests that the social work workforce is characterized by high mobility and relatively low occupational stability.

Monthly income ranged from CNY 800 to 10,000, reflecting generally low income levels with notable disparity. The largest proportion of respondents earned between CNY 2,001 and 3,000; only 3.3% reported a monthly income exceeding CNY 5,000.

### 3.3 Measurement

#### 3.3.1 Independent constructs

This study examines the influence of working conditions on turnover intention. Drawing on the measurement of working conditions in the PM and JD-R models and the structure of the CSWLS 2019 questionnaire, we operationalized Job Stress, Work Support, and Salary as proxy variables for working conditions.

Job stress is measured by four items based on the PM models (Price, 2001): “I lack sufficient resources to complete my work tasks”(H1F); “I perform tasks that are not very necessary”(H1G); “The organization lacks rules and regulations to assist me in my work”(H1H); and “The organization's rules and regulations contain conflicting elements”(H1I).

Work support is composed of four dimensions according to (Comer et al. 1997): support from direct supervisor (S1), department manager (S2), peers (S3), and top leadership (S4). Six identical items measure each dimension (Comer et al., 1997): “They provide reliable support when I encounter difficulties”; “They are willing to listen to my work problems”; “They are willing to help me complete my work”; “They possess professional competence”; “They can handle their own tasks well”; and “They specifically praise employees for outstanding performance.” The score for each dimension is the mean of its six items.

Salary. Measured by the single item (D18): “What is your current monthly take-home pay?”

#### 3.3.2 Dependent construct

The dependent variable in this study is Turnover intention, measured by three items (Li et al., 2022; Yuan et al., 2021): “I plan to leave my current organization within the next 6 months (D39A),” “I may leave my current organization within the next 3 years (D39B),” and “I occasionally consider leaving my current organization (D39C).”

#### 3.3.3 Mediating constructs

The mediating variables are job satisfaction and job embeddedness. Each dimension's value represents the mean score of its items.

Job satisfaction is measured by five items (Yuan et al., 2021): “I find real enjoyment in my work” (L1A); “My job is unusual” (L1B); “I love my work more than the average person” (L1C); “Most of the time, I am enthusiastic about my work” (L1E); and “I am quite satisfied with my job” (L1G).

Regarding job embeddedness, (Mitchell et al. 2001) conceptualized it as a formative construct comprising three dimensions: “Links,” “Fit,” and “Sacrifice”. According to Mitchell et al., “Links” is measured by five items regarding whether key social connections support staying: “My elders (D40A)/friends (D40C)/colleagues (D40D)/leaders (D40E)/people who are important to me (D40F) believe that I should stay in my current job.” “Fit” is measured by three items: “I identify with the organizational culture” (G1E), “I identify with the organization's values” (G1G), and “My values align with those of most members” (G1H). “Sacrifice” is measured by four items assessing potential gains from leaving: “If I left, I would take a job with better pay (P1A)/greater achievement (P1B)/more advancement (P1F)/or higher status (P1H)”. These items were reverse-coded prior to analysis (e.g., 5 recoded as 1) so that higher scores indicate higher perceived costs of leaving.

#### 3.3.4 Control variables

The covariates in this study primarily include the demographic characteristics of the respondents: age (Age), gender (A1), educational background (B1), marital status (A9) and the respondent's tenure in the organization (T1).

### 3.4 Method

This study aims to explore the mechanisms by which working conditions influence turnover intention, with a specific focus on the mediating effects of job satisfaction and job embeddedness. Regarding data analysis, SPSS 27.0 was utilized for data cleaning and descriptive statistics. Owing to the variables in this study being latent constructs, we are advised to use Structural Equation Modeling (SEM) to test the relationship between them (Sarstedt et al., 2019). SEM was utilized because it explicitly accounts for measurement error in observed items, thereby providing more accurate estimates of relationships between latent constructs than traditional regression techniques. Furthermore, SEM allows simultaneous estimation of the measurement model (relationships between items and constructs) and the structural model (relationships among constructs), thereby ensuring rigorous assessment of both construct validity and theoretical hypotheses. Compared to Covariance-Based SEM (CB-SEM), Partial Least Squares Structural Equation Modeling (PLS-SEM) is particularly suitable for models incorporating both reflective and formative constructs, and research aimed at exploring or extending existing theories (Hair et al., 2019). In this study, “Job Embeddedness” is conceptualized as a reflective-formative second-order construct; we therefore employed SmartPLS 4 to conduct PLS-SEM (Sarstedt et al., 2019).

Similar to CB-SEM, PLS-SEM evaluation involves assessing both the measurement and the structural models.

Since “Job Embeddedness” is a higher-order construct, the data analysis followed the two-stage approach proposed by (Sarstedt et al. 2019). Currently, the mainstream methods are the disjoint two-stage approach (Becker et al., 2012) and the embedded two-stage approach (Ringle et al., 2020). Prior research indicates that although these methods differ in configuration, they yield negligible differences in results (Cheah et al., 2019). This study adopts the Disjoint Two-Stage Approach.

**Figure 3:**
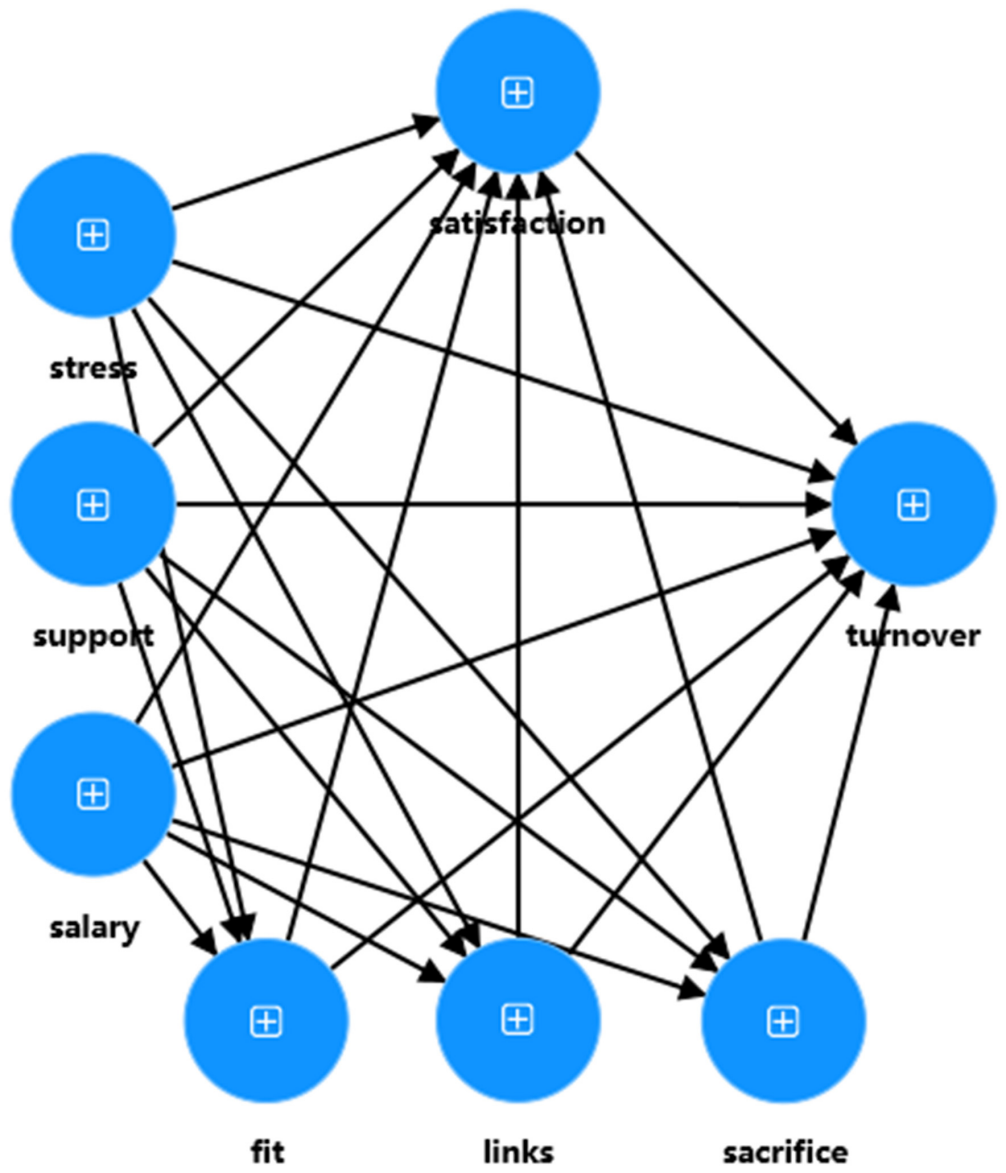
The model of stage 1.

**Figure 4:**
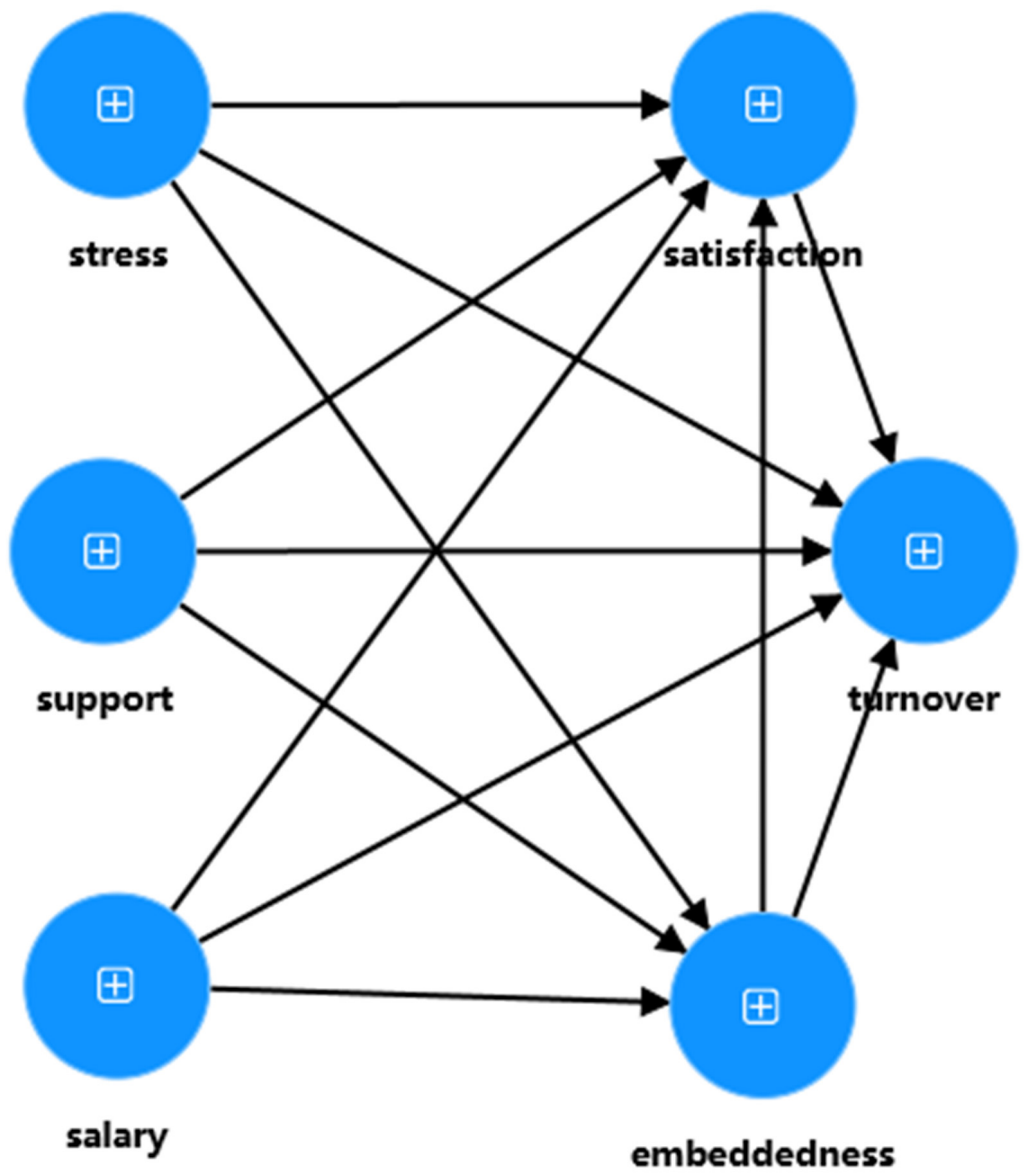
The model of stage 2.

In stage 1, the three dimensions (“Links,” “Fit,” and “Sacrifice”) that make up job embeddedness are treated as separate variables in the model analysis. The purpose of this stage is to assess the measurement model and obtain latent construct scores for the 3 lower-order constructs. In stage 2, the latent variable scores obtained in Stage 1 are used to replace the original dimensions in the model. The purpose of this stage is to evaluate the structural model and test the proposed hypotheses. Figures 3, 4 illustrate the models for stage 1 and stage 2 of this study, respectively.

## 4 Results

### 4.1 Descriptive statistics

The descriptive statistics of the core variables are summarized in Table 1. We found that the mean scores for items measuring job stress were generally in the low-to-moderate range (M = 2.67–3.07). Scores for items measuring work support were relatively high (M = 3.75–4.10); specifically, “Supervisor Support” (M = 4.10) and “Peer Support” (M = 4.09) reached high levels, while “Top Leadership Support” was at an upper-moderate level (M = 3.75). Regarding salary level, the average monthly take-home pay for social workers was CNY 3.22 thousand (SD = 1.10), indicating a generally low overall income level with substantial distributional disparity.

**Table 1:** Descriptive statistics of core construct (*n* = 671).

**Variables**	**Measurement**	**M**	**SD**	**Unit definition**	**Source**
Stress	H1F	3.07	0.898	1–5 Likert scale	Price, 2001; Yuan et al., 2021
	H1G	2.97	0.998	1–5 Likert scale	
	H1H	2.79	0.926	1–5 Likert scale	
	H1I	2.67	0.892	1–5 Likert scale	
Work support	S1	3.94	1.07	1–5	Comer et al., 1997
	S2	4.10	0.97	1–5	
	S3	4.09	0.86	1–5	
	S4	3.75	1.16	1–5	
Salary	D18	3.22	1.10	0.8–10 (Thousand, CNY)	/
Links	D40A	2.86	1.05	1–5 Likert scale	Mitchell et al., 2001
	D40C	2.87	0.96	1–5 Likert scale	
	D40D	3.22	0.89	1–5 Likert scale	
	D40E	3.5	0.91	1–5 Likert scale	
	D40F	3.07	0.93	1–5 Likert scale	
Fit	G1E	3.78	0.76	1–5 Likert scale	
	G1G	3.88	0.72	1–5 Likert scale	
	G1H	3.8	0.69	1–5 Likert scale	
Sacrifice	P1A(reverse)	2.16	0.89	1–5 Likert scale	
	P1B(reverse)	2.38	0.87	1–5 Likert scale	
	P1F(reverse)	2.3	0.85	1–5 Likert scale	
	P1H(reverse)	2.55	0.83	1–5 Likert scale	
Job satisfaction	L1A	3.64	0.73	1–5 Likert scale	Yuan et al., 2021
	L1B	3.44	0.82	1–5 Likert scale	
	L1C	3.44	0.80	1–5 Likert scale	
	L1E	3.82	0.69	1–5 Likert scale	
	L1G	3.41	0.82	1–5 Likert scale	
Turnover intenstion	D39A	2.17	1.05	1–5	Li et al., 2022; Yuan et al., 2021
	D39B	2.87	1.07	1–5	
	D39C	2.97	1.10	1–5	

Among the three dimensions of job embeddedness, items measuring the “Fit” dimension generally scored high (M = 3.78–3.88). Items measuring the “Links” dimension showed moderate overall levels (M = 2.86–3.50). Items measuring the “Sacrifice” dimension scored generally low (M = 2.16–2.55).

Scores on items measuring job satisfaction were in the upper-moderate range (M = 3.41–3.82).

Regarding turnover intention, short-term intention to leave was low (M = 2.17); however, the intention to potentially leave within the next 3 years (M = 2.87) and occasional thoughts of leaving (M = 2.97) both approached the moderate threshold.

In summary, regarding organizational working conditions, the surveyed social workers experienced relatively low job stress and high Work support, but low salary levels. As to job embeddedness, they exhibited moderate levels of “Links”, high levels of “Fit”, and low levels of “Sacrifice.” Regarding job satisfaction, the overall level was favorable. In terms of turnover intention, social workers' intentions to leave were in the medium- to long-term.

### 4.2 Measurement model assessment

#### 4.2.1 Reflective measurement models

In the first stage, the dimensions constituting job embeddedness—“Links,” “Sacrifice,” and “Fit”—were incorporated into the analysis as individual reflective measurement models. Reliability was assessed by Cronbach's α and Composite Reliability (CR), while convergent validity was evaluated by Outer Loadings (OL) and Average Variance Extracted (AVE). Discriminant validity was examined using the Heterotrait-Monotrait ratio (HTMT) (Hair, 2014; Hair Jr et al., 2020).

During the initial analysis of Outer Loadings, items “D40E” (used to measure “Links”) and “H1F” (used to measure “Stress”) yielded loadings of 0.636 and 0.614, respectively. As values fell below the critical threshold of 0.70 (Hair et al., 2022), these items were removed.

Upon re-analysis following the exclusion of items with insufficient loadings, the results indicated that Cronbach's α for all constructs exceeded 0.70, and CR values exceeded 0.80. Both metrics surpassed the critical threshold of 0.60 (Cronbach, 1951; Werts et al., 1974), demonstrating robust internal consistency and measurement reliability. Furthermore, the AVE for all constructs exceeded 0.60, surpassing the 0.50 threshold (Fornell and Larcker, 1981), and all item outer loadings exceeded 0.70. These findings confirm that all constructs possess satisfactory convergent validity. Table 2 presents the assessment of the reflective measurement model.

**Table 2:** Assessment of the reflective measurement model.

**Construct/dimension**	**Measurement**	**Outer loading**	**Cronbach α**	**CR**	**AVE**
Fit	G1E	0.92	0.88	0.93	0.80
	G1G	0.92			
	G1H	0.84			
Links	D40A	0.82	0.86	0.90	0.70
	D40C	0.88			
	D40D	0.74			
	D40F	0.89			
Sacrifice	P1Areverse	0.86	0.85	0.90	0.68
	P1Breverse	0.81			
	P1Freverse	0.86			
	P1Hreverse	0.77			
Satisfaction	L1A	0.83	0.86	0.90	0.63
	L1B	0.73			
	L1C	0.83			
	L1E	0.78			
	L1G	0.81			
Stress	H1G	0.77	0.76	0.86	0.68
	H1H	0.89			
	H1I	0.81			
Support	S1	0.84	0.86	0.90	0.70
	S2	0.88			
	S3	0.78			
	S4	0.85			
Turnover	D39A	0.78	0.76	0.86	0.67
	D39B	0.86			
	D39C	0.82			

Regarding discriminant validity, Table 3 presents the HTMT results. The maximum HTMT value between variables was 0.486, well below the stringent threshold of 0.90 (Hair et al., 2022). Additionally, the Fornell-Larcker criterion showed that the square root of the AVE for each construct was greater than its correlation with any other construct. The cross-loadings analysis also confirmed that each item loaded higher on its assigned construct than on any other. Thus, we conclude that the variables are statistically distinct, and the measurement model demonstrates robust discriminant validity.

**Table 3:** Assessment of discriminant validity using the HTMT.

**Construct/dimension**	**Fit**	**Links**	**Sacrifice**	**Satisfaction**	**Stress**	**Support**
Links	0.27	/	/	/	/	/
Sacrifice	0.11	0.24	/	/	/	/
Satisfaction	0.45	0.25	0.10	/	/	/
Stress	0.44	0.21	0.19	0.39	/	/
Support	0.42	0.17	0.09	0.34	0.49	/
Turnover	0.36	0.46	0.37	0.46	0.49	0.35

Values represent the Heterotrait-Monotrait (HTMT) indices for the constructs/dimensions corresponding to the rows and columns.

### 4.3 Formative measurement model

In the second stage, the dimensions “Links,” “Fit,” and “Sacrifice” were proxied by latent variable scores calculated using the Importance-Performance Map Analysis (IPMA). Job Embeddedness was operationalized as a formative first-order model.

This study evaluated the validity of the Job Embeddedness construct based on three criteria: (1) the Variance Inflation Factor (VIF) values among the three dimensions; (2) the correlation and significance between the dimensions and the Job Embeddedness construct; and (3) the outer weights and their significance.

Due to the absence of a global single item for Job Embeddedness (which prevents standard redundancy analysis), we followed the approach of previous studies (Le et al., 2022; Luo et al., 2012; Petter et al., 2007). We calculated a weighted score for each dimension by multiplying the latent variable score by its weight. Then we summed these weighted scores to obtain a composite score for job embeddedness. We then examined the correlations between the weighted scores of each dimension and the composite score. Table 4 presents the assessment results for the formative measurement model.

**Table 4:** Assessment of formative measurement model.

**Construct**	**Dimension**	**Outer weight**	**Correlation**	**VIF**
Embeddedness	Fit	0.759^***^	0.877^***^	1.064
	Links	0.405^***^	0.632^***^	1.103
	Sacrifice	0.212^**^	0.368^***^	1.048

Significance levels: ^*^P < 0.05, ^**^P < 0.01, ^***^P < 0.001.

#### 4.3.1 Collinearity

The VIF values for all dimensions were well below the strict threshold of “3” (Hair et al., 2011), indicating the absence of severe multicollinearity issues among the indicators.

#### 4.3.2 Convergent validity

All dimensions were significantly correlated with the Job Embeddedness construct, indicating satisfactory convergent validity.

#### 4.3.3 Weights significance

The outer weights for all dimensions were significant, indicating that each dimension makes a statistically significant contribution to the higher-order construct of job embeddedness.

Furthermore, (Mitchell et al. 2001) posit that job embeddedness should significantly and negatively predict turnover intention. To further assess the criterion validity of the job embeddedness measurement model, we analyzed its relationship with turnover intention. The results revealed a significant negative correlation (*r* = −0.448, *t* = 10.625, *p* < 0.001), indicating that the measurement model constructed in this study has robust validity and meets theoretical expectations.

So, the dimensions of “Links,” “Fit,” and “Sacrifice” effectively form the Job Embeddedness construct.

### 4.4 Structural model assessment

Unlike CB-SEM, which emphasizes overall model fit, PLS-SEM focuses on assessing the explanatory power of the endogenous constructs within the structural model. The assessment primarily consists of five parts: collinearity assessment, model fit assessment, path coefficient assessment, explanatory power assessment (R^2^), and predictive power assessment (Q^2^) (Hair et al., 2022).

#### 4.4.1 Collinearity and model fit assessment

In PLS-SEM, path coefficients are estimated via Ordinary Least Squares (OLS) regressions of each endogenous latent variable on its corresponding predictors, a process that is potentially susceptible to multicollinearity. To assess collinearity, this study used the Variance Inflation Factor (VIF) for diagnostic purposes. The results indicate that the maximum VIF value among all indicators was 2.721, which is below the threshold of “3”, demonstrating the absence of collinearity issues among the indicators (Hair et al., 2011).

Currently, the evaluation of model fit in PLS-SEM is a subject of considerable debate within the academic community. (Hair et al. 2022) argue that PLS-SEM prioritizes prediction over explanatory modeling. Furthermore, the indices currently used to test PLS-SEM fit—such as SRMR, d_ULS, d_G, Chi2, NFI—are originally designed for CB-SEM. (Sarstedt et al. 2016) suggest that the aforementioned indices should be treated as heuristic criteria (reference indicators) rather than mandatory criteria, with results serving only as a reference. So, we adopt SRMR as the reference metric for assessing model fit. It is generally accepted that an SRMR value below 0.08 indicates good model fit (Henseler et al., 2016). The structural equation model constructed in this study yielded an SRMR of 0.061, indicating satisfactory goodness of fit.

#### 4.4.2 Assessment of effects

The assessment of effects (total, direct, and indirect) is central to the evaluation of the PLS-SEM structural model (Hair et al., 2022). The Bootstrapping method with 5,000 resamples was employed to generate the sampling distribution of coefficient estimates and calculate 95% confidence intervals (CI) to determine significance. Tables 5–7 present the results for the total, direct, and indirect effects, respectively.

**Table 5:** Results of total effects.

**Path**	**β**	***t***	**95% confidence intervals (CI)**
Stress -> turnover	0.273^***^	7.212	(0.195, 0.353)
Support -> turnover	−0.215^***^	6.352	(−0.284, −0.150)
Salary -> turnover	−0.113^**^	2.872	(−0.191, −0.035)
Embeddedness -> turnover	−0.298^***^	7.091	(−0.382, −0.217)
Satisfaction -> turnover	−0.141^**^	3.374	(−0.221, −0.055)
Age -> turnover	−0.094^*^	2.060	(−0.184, −0.005)
Tenure -> turnover	−0.139^***^	3.956	(−0.209, −0.070)
Education -> turnover	0.085^*^	20,295	(0.013, 0.157)
Gender -> turnover	−0.021	0.232	(−0.194, 0.154)
Marriage -> turnover	−0.198^*^	2.460	(−0.354, −0.033)

Significance levels: ^*^P < 0.05, ^**^P < 0.01, ^***^P < 0.001.

**Table 6:** Results of direct effects.

**Path**	**β**	***t***	**95% confidence intervals (CI)**
Stress -> turnover	0.177^***^	4.519	(0.100, 0.254)
Support -> turnover	−0.110^**^	3.004	(−0.184, −0.041)
Salary -> turnover	−0.078^*^	2.074	(−0.152, −0.004)
Embeddedness -> turnover	−0.264^***^	6.124	(−0.351, −0.182)
Satisfaction -> turnover	−0.141^**^	3.374	(−0.221, −0.055)
Age -> turnover	−0.036	0.857	(−0.018, −0.045)
Tenure -> turnover	−0.129^***^	3.829	(−0.197, −0.062)
Education -> turnover	0.048	1.370	(−0.021, 0.120)
Gender -> turnover	−0.039	0.465	(−0.2000, 0.124)
Marriage -> turnover	−0.170^*^	2.291	(−0.310, −0.020)

The values reported in the table are standardized path coefficients (β). Values in parentheses are 95% confidence intervals (CI). Significance levels: ^*^P < 0.05; ^**^P < 0.01; ^***^P < 0.001.

**Table 7:** Results of indirect effects.

**Path**	**β**	**t**	**95% CI**
Stress -> embeddedness -> turnover	0.065^***^	4.007	(0.036, 0.101)
Stress -> satisfaction -> turnover	0.022^*^	2.388	(0.006, 0.042)
Stress -> embeddedness -> satisfaction -> turnover	0.009^*^	2.54	(0.003, 0.016)
Support -> embeddedness -> turnover	−0.072^***^	4.666	(−0.105, −0.045)
Support -> satisfaction -> turnover	−0.024^*^	2.559	(−0.044, −0.008)
Support -> embeddedness -> satisfaction -> turnover	−0.009^*^	2.511	(−0.018, −0.003)
Salary -> embeddedness -> turnover	−0.011	1.193	(−0.032, 0.005)
Salary -> satisfaction -> turnover	−0.022^**^	2.667	(−0.039, −0.007)
Salary -> embeddedness -> satisfaction -> turnover	−0.001	1.098	(−0.005, 0.001)

The reported indirect effects refer to the hypothesized mediation paths. For the sake of brevity, the indirect effects associated with control variables are omitted. Significance levels: ^*^P < 0.05; ^**^P < 0.01; ^***^P < 0.001.

#### 4.4.3 The impact of organizational working conditions on turnover intention

Path analysis examined the effect of internal working conditions on turnover intention. Regarding job stress, the total effect shows job stress significantly and positively predicts Turnover Intention (β = 0.273, *p* < 0.001). After controlling for mediators, job stress still directly and significantly affects turnover intention (β = 0.177, *p* < 0.001, 95% CI). In terms of work support, the total effect shows that work support significantly and negatively influences turnover intention (β = −0.215, *p* < 0.001, 95% CI). After controlling for mediators, work support still directly and negatively influences turnover intention (β = −0.11, *p* < 0.01, 95% CI). As to salary, the total effect analysis shows that it significantly and negatively influences turnover intention (β = −0.113, *p* < 0.01, 95% CI). After controlling for mediators, salary still directly and negatively influences turnover intention (β = −0.078, *p* < 0.05, 95% CI).

#### 4.4.4 Mediation analysis

First of all, job stress not only significantly increases turnover intention by reducing job embeddedness (β = 0.065, *p* < 0.001, 95% CI) but also by reducing job satisfaction (β = 0.022, *p* < 0.05, 95% CI). Moreover, the serial mediation path (job stress → job embeddedness → job satisfaction → turnover intention) is significant (β = 0.009, *p* < 0.001, 95% CI).

Secondly, work support not only significantly reduces turnover intention by increasing job embeddedness (β = −0.072, *p* < 0.001, 95% CI) but also by increasing job satisfaction (β = −0.024, *p* < 0.05, 95% CI). In addition, the serial mediation path (work support → job embeddedness → job satisfaction → turnover intention) significantly inhibits turnover intention (β = −0.009, *p* < 0.05, 95% CI).

Thirdly, the mediation analysis shows that salary only indirectly and significantly affects turnover intention via job satisfaction (β = −0.022, *p* < 0.01, 95% CI), and the paths “salary → job embeddedness → turnover intention” and “salary → job embeddedness → job satisfaction → turnover intention” were not significant.

#### 4.4.5 Explanatory and predictive power

Evaluating the model's explanatory and predictive power is also crucial (Hair et al., 2022). According to (Hair et al. 2019), R^2^ (coefficient of determination) measures the proportion of variance in the endogenous latent variable that the model explains. f^2^ (effect sizes) quantifies the contribution of a specific exogenous variable to the explained variance of an endogenous variable. Both are used to measure explanatory power. Q^2^ assesses the model's predictive relevance. Table 8 reports the variance explained (R^2^) and predictive relevance (Q^2^), and Table 9 reports the effect size (f^2^) results.

**Table 8:** Assessment of effect size using the R^2^ and Q^2^.

**Construct**	**R^2^**	**Q^2^**
Embeddedness	0.25	0.09
Satisfaction	0.26	0.15
Turnover	0.35	0.22

**Table 9:** Assessment of effect size using the f^2^.

**Construct**	**Embeddedness**	**Satisfaction**	**Turnover intention**
Stress	0.066	0.025	0.035
Support	0.080	0.029	0.014
Salary	0.002	0.027	0.008
Embeddedness	/	0.060	0.075
Satisfaction	/	/	0.023

As shown in Table 8, the model's R^2^ value exceeds 0.25, indicating that the model possesses a certain degree of explanatory power. Furthermore, the Q^2^ values for all endogenous variables are greater than 0, providing robust evidence that the parameter estimates can be effectively applied to predict new sample data, thus supporting the model's external validity.

Table 9 shows that the f^2^ values for job stress, job embeddedness, and job satisfaction on turnover intention all exceed 0.02, indicating that while their degrees of influence may vary, these variables make statistically discernible and unique explanatory contributions to turnover intention, suggesting their theoretical significance should not be overlooked in practice. Conversely, the f^2^ values for Salary and Work Support on Turnover Intention are less than 0.02, signifying that the overall explanatory power of these variables for turnover intention is weak and their practical impact is relatively limited (Hair et al., 2019).

### 4.5 Robustness check

Robustness testing in PLS-SEM is a critical but often overlooked component. According to (Sarstedt et al. 2020), the robustness check for PLS-SEM includes 3 parts. The first part is the nonlinear effects check, the second is the endogeneity check, and the last is the unobserved heterogeneity check.

#### 4.5.1 Nonlinear effects

For nonlinear effects, (Sarstedt et al. 2019) suggest that researchers can run Ramsey's (1969) regression equation specification error test (RESET) to assess whether the relationships among constructs in their model are nonlinear. The Assessment of nonlinear effects in Table 10 shows that the partial regressions of Embeddedness, Satisfaction, and Turnover are not subject to nonlinearities.

**Table 10:** Assessment of nonlinear effects.

**Dependent construct**	**Independent construct**	**F**	***P*-value**
Embeddedness	Support Stress Salary	F(3,659) *=* 0.12	0.95
Satisfaction	Support Stress Salary Embeddedness	F(3,658) *=* 0.69	0.56
Turnover intention	Support Stress Salary Embeddedness Satisfaction	F(3,657) *=* 1.64	0.18

#### 4.5.2 Endogeneity

In PLS-SEM, endogeneity occurs when the independent variable is correlated with the error term of the dependent variable (Bascle, 2008). So, the endogeneity in PLS-SEM arises mainly from omitted variables. (Hult et al. 2018) developed a systematic procedure for checking and treating the endogeneity in PLS-SEM. The first step is to detect endogeneity using the Gaussian copula approach. If this approach indicates endogeneity, we first need to add control variables available to address it. If adding additional control variables does not work, researchers are advised to use instrumental variables to address endogeneity. In Table 11, we find that none of the Gaussian copulas is significant (*P* > 0.05). We therefore conclude that endogeneity is not a critical concern in this study.

**Table 11:** Assessment of endogeneity test using the Gaussian copula approach.

**Model**	**Endogenous variable**	**Cofficient of Gaussian copula**	**P-value of Gaussian copula**
Gaussian copula of model 1	Support	−0.11	0.148
Gaussian copula of model 2	Stress	−0.05	0.907
Gaussian copula of model 3	Salary	−0.04	0.657
Gaussian copula of model 4	Embeddedness	−0.15	0.595
Gaussian copula of model 5	Satisfaction	0.26	0.244

#### 4.5.3 Unobserved heterogeneity

Finite mixture PLS approach is used to ascertain whether the heterogeneity is a problem. The results of a post hoc power analysis assuming an effect size of 0.15 and a power level of 80% suggest that the minimum sample size requirement is 85 (Sarstedt et al., 2020). We have 671 samples so that we can rerun FIMIX-PLS for 2 to 7 segments. (Sarstedt et al. 2011) indicated that researchers should jonit consider modified Akaike's information criterion with factor 3 (AIC3) and consistent AIC (CAIC) after evaluating the efficacy of various model selection criteria. AIC3 and CAIC in Table 12 point to different segment numbers, indicating the analyses do not unambiguously point to a specific segmentation solution. So, we can assume that unobserved heterogeneity is not at a critical level.

**Table 12:** Fit indices for the one- to seven-segment solutions.

**Segment**	**AIC3**	**CAIC**
1	5,131.573	**5,266.836**
2	5,087.677	5,362.712
3	5,072.042	5,486.849
4	5,060.241	5,614.820
5	5,067.087	5,761.438
6	5,044.437	5,878.559
7	**4,987.831**	5,961.725

Bold values indicate the best performance in each segment.

Having rigorously addressed potential concerns related to nonlinearity, endogeneity, and unobserved heterogeneity, we confirm that the study's PLS-SEM results remain robust.

## 5 Discussion and conclusion

### 5.1 Discussion

This study employed PLS-SEM to examine the influence of organizational working conditions on social workers' turnover intention, focusing on the mediating roles of job satisfaction and, especially, job embeddedness in the relationship between working conditions and turnover intention. The findings validate the constructed model. The specific discussions are as follows.

#### 5.1.1 Organizational working conditions significantly affect social workers' turnover intention

Regarding job stress, it significantly and directly predicts a positive increase in social workers' turnover intention, aligning with findings by (Li et al. 2025) and (Wooten et al. 2011) (H1a supported). According to (Hobfoll 1989), individuals constantly accumulate resources for survival, and stress continuously depletes them. When resources are overdrawn due to stress, individuals opt for turnover to halt the resource loss.

In terms of work support, it significantly and directly reduces social workers' turnover intention, consistent with studies by (Mor Barak et al. 2001) and (Karatepe 2013) (H1b supported). The comprehensive protective role of work support aligns with Social Exchange Theory (Blau, 1964): when organizations provide support, employees perceive organizational commitment and care, generating an obligation to reciprocate. This norm of reciprocity directly strengthens the intention to remain.

As for salary, it significantly and directly reduces turnover intention, consistent with the findings of (Hanifa et al. 2024) and (Luo and Chui 2020) (H1c supported). From the “rational economic man” perspective, individuals maximize their economic self-interest, making salary level an undeniable determinant of employee retention.

#### 5.1.2 Job embeddedness played a crucial and significant mediating role

##### 5.1.2.1 Single mediation effects

Job embeddedness significantly mediated the effects of job stress and work support on turnover intention (H3a and H3b supported). However, its mediation was not significant in the relationship between salary and turnover intention (H3c not supported). Job satisfaction served as a significant mediator across all three relationships (job stress, work support, and salary) (H2a, H2b, and H2c supported).

A possible explanation for the non-significance of H3c is that salary more directly fulfills employees' survival and equity needs, boosting job satisfaction, than it influences job embeddedness. Job embeddedness is a deep, multidimensional construct covering “Links,” “Match,” and “Sacrifice.” Therefore, simple salary increases are insufficient to shape the complex, deep social connections between the employee and the organization.

##### 5.1.2.2 Serial mediation effects

The serial mediating role of job embeddedness and job satisfaction was significant in the paths involving job stress and work support (H4a and H4b supported). This effect was not significant in the salary path (H4c not supported).

The establishment of this serial mediation in most paths validates a progressive influence mechanism for employee turnover intention: external factors first affect the employee's structural embeddedness, and this sense of embeddedness fosters positive affective evaluation of the job, which then translates into lower turnover intention. This chain illustrates that retention is a systematic effort requiring comprehensive intervention, ranging from improving the work environment to deepening organizational belonging, and finally to enhancing the work experience. The failure of this chain in the salary path likely stems from the non-significant link between salary level and job embeddedness.

##### 5.1.2.3 The superior explanatory power of job embeddedness

Crucially, the influence of job embeddedness on social workers‘ turnover intention was stronger than that of job satisfaction. The mediation tests showed that the mediating effect of job embeddedness in the paths from job stress and work support to turnover intention was significantly stronger than that of job satisfaction. Furthermore, the f^2^ index indicated that job embeddedness contributed more to explaining the variance in turnover intention than did job satisfaction. This finding highlights the critical status of job embeddedness in the turnover model and validates the theoretical innovation of incorporating it into the mediating mechanism. Social work is fundamentally a relationship-based practice. The strong interpersonal connections that social workers establish with both clients and colleagues serve as critical resources that facilitate their retention (Trevithick, 2008). Moreover, leaving the organization brings a high psychological cost, as it implies forfeiting the professional identity of a “helper” and abandoning substantial emotional investments (Strolin-Goltzman, 2010). Social workers' personal values, such as altruism and justice, are often closely aligned with their agency's mission, which stems from professional values and principles (Giffords, 2009).

Finally, control variables, including age, education level, marital status, and tenure, also had significant effects on the mediating variables and turnover intention, emphasizing the non-negligible role of individual characteristics in turnover decisions.

### 5.2 Conclusion

In summary, this study develops and integrates the PM and JD-R models, making the theoretical framework more suitable for explaining social workers‘ turnover intention by adding the construct of “job embeddedness” as a critical mediator. The proposed model demonstrates good overall fit, possesses reliable predictive and explanatory power, and shows that the core variables make significant contributions to explaining turnover intention, offering both theoretical significance and practical implications. Based on these results, we propose several recommendations to assist organizations in optimizing their management strategies and effectively mitigating social workers' turnover intention.

#### 5.2.1 Optimization of internal working conditions

First, alleviate job stress. Organizations must establish robust mechanisms for psychological support and stress management. Furthermore, management should reduce role ambiguity and work overload by rationally allocating tasks and clearly defining role boundaries.

Then, strengthen work support. Organizations should enhance work support through team building and the development of peer support networks. Special attention should be paid to employees in high-stress positions to foster an inclusive climate of mutual assistance.

Last but not least, refine the compensation system. To enhance motivation, organizations should establish a fair, transparent, and competitive compensation system by integrating with a multi-dimensional incentive structure that includes both performance-based pay and non-material rewards.

#### 5.2.2 Prioritizing job embeddedness in retention strategies

When addressing turnover, organizations must prioritize job embeddedness. Our results indicate that job embeddedness possesses greater explanatory power for turnover intention than job satisfaction. Therefore, organizations should focus on the three dimensions of embeddedness (“Links,” “Fit,” and “Sacrifice”) to strengthen the employee-organization bond.

First, enhancing “Links.” Organizations should systematically construct a relational network that tightly binds employees to the organization. When employees perceive that leaving requires severing numerous ties, they are more inclined to stay. Increase interaction and mutual aid across different departments and colleagues. Strengthen supervisor support and enhance communication between upper management and subordinates.

Second, improving “Fit.” Organizations must ensure employees perceive high compatibility between their personal values, skills, and lifestyles and the organization's culture and job requirements. Use value assessment and cultural fit tools to select candidates aligned with core organizational beliefs. Implement personalized career paths and continuous training to achieve a dynamic match between personal growth and job demands, thereby enhancing professional belonging. Cultivate an inclusive atmosphere that respects diversity and personal life needs, fostering harmony between the individual, the organization, and the community.

Third, increasing “Sacrifice.” To heighten the perceived cost of leaving, organizations should establish a value system that makes “disembedding” difficult by creating non-portable benefits that employees would lose upon exit, such as tenure-based welfare packages and unique organizational honors.

#### 5.2.3 Differentiated human resource management

Finally, organizations must account for individual differences. Prior research has revealed that individual factors, such as age, gender, and personality traits, significantly influence various facets of social workers' professional lives and job attitudes (Mor Barak et al., 2001). Consequently, it is crucial to recognize and address the heterogeneity within the social work workforce. Differentiated management and support strategies should be implemented based on employees' varying demographic characteristics and work experiences. This approach could help human resource policies become both targeted and effective, thereby significantly mitigating social workers' turnover intention.

### 5.3 Limitations and future study

#### 5.3.1 Limitations

However, this study has certain limitations. First, restricted by the availability of secondary data and our specific focus on the organizational environment, this study examines only the relationship between organizational embeddedness, working conditions, and turnover intention. This inevitably leads to the exclusion of community embeddedness, despite its recognized importance in social work. Second, the cross-sectional design limits our ability to draw strict causal inferences between variables. Third, given that the data were collected in 2019 exclusively within China, the findings may have temporal and geographical constraints regarding their generalizability to other cultural contexts or the current era.

#### 5.3.2 Future study

Based on the limitations discussed above, we propose several directions for future research. First, future studies should incorporate community embeddedness into the model to provide a holistic view of how both 'on-the-job' and 'off-the-job' forces jointly influence social workers' retention. Second, to enhance external validity, scholars are encouraged to validate this model across diverse cultural contexts. Finally, given the cross-sectional nature of this study, we suggest employing longitudinal designs to rigorously establish the causal directions between working conditions, job embeddedness, job satisfaction, and turnover intention.

## Data Availability

Publicly available datasets were analyzed in this study. This data can be found here: The data presented in this study are available in the China Social Work Longitudinal Study 2019 (CSWLS2019) repository at https://mp.weixin.qq.com/s/kOQTkzQK7e2wN5WOMQIOvw.
