# Supplementary material for: Beyond job satisfaction: a job embeddedness-based mediation model to explain turnover intention in Chinese social workers
Source: Front Psychol. 2026 Feb 20;17:1766949. doi: 10.3389/fpsyg.2026.1766949 (PMC12962906; doi:10.3389/fpsyg.2026.1766949)
Supplement: Supplementary file 1 [file Table_1.DOCX]

Supplementary Material

# Supplementary Figures and Tables


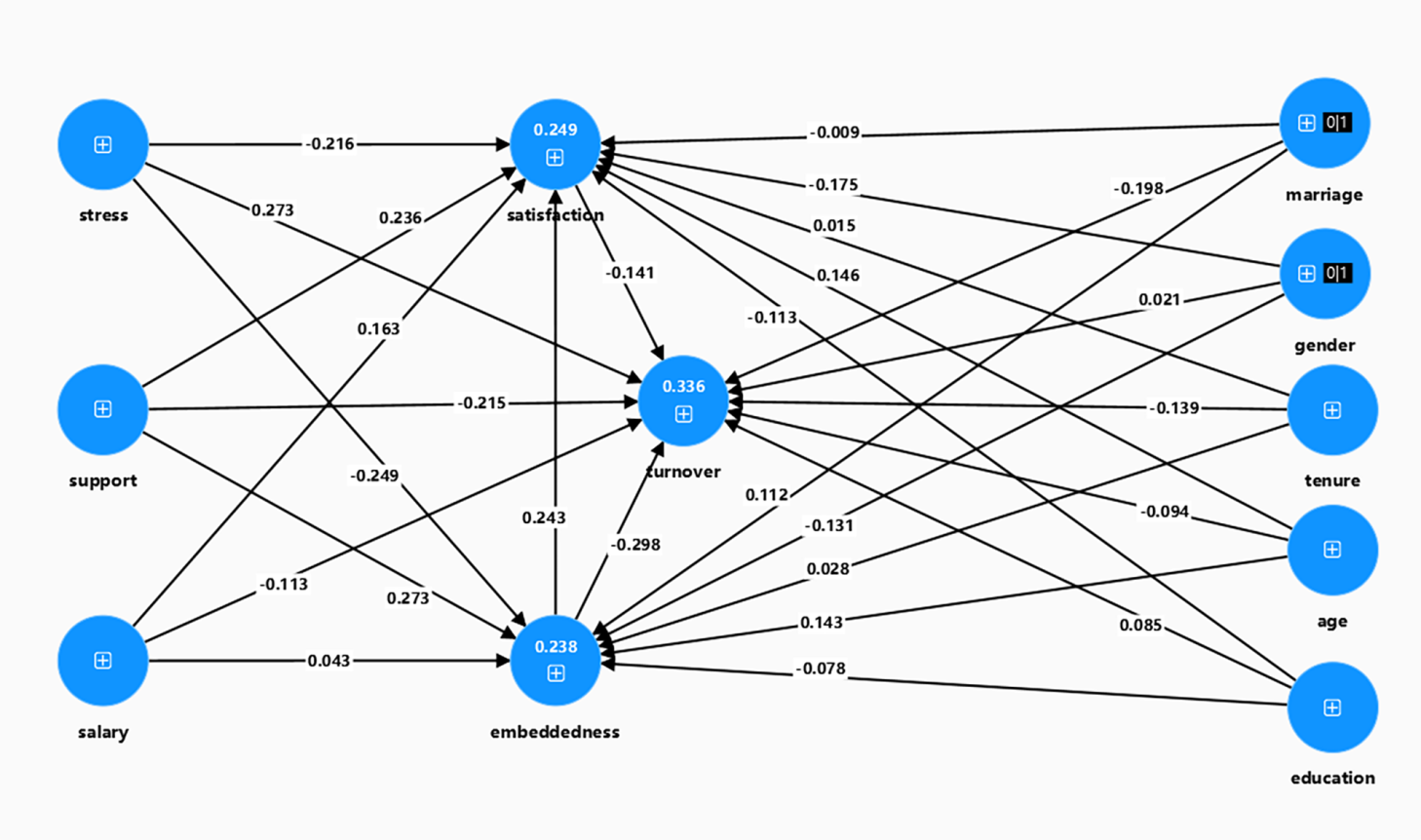


***Note.*** *Values on the arrows represent standardized path coefficients. Values inside the blue circles indicate the adjusted coefficient of determination (R^2^) for embeddedness, satisfaction, and turnover intention.*

**Supplementary Figure 1.** Total effects of the proposed model (SmartPLS output).


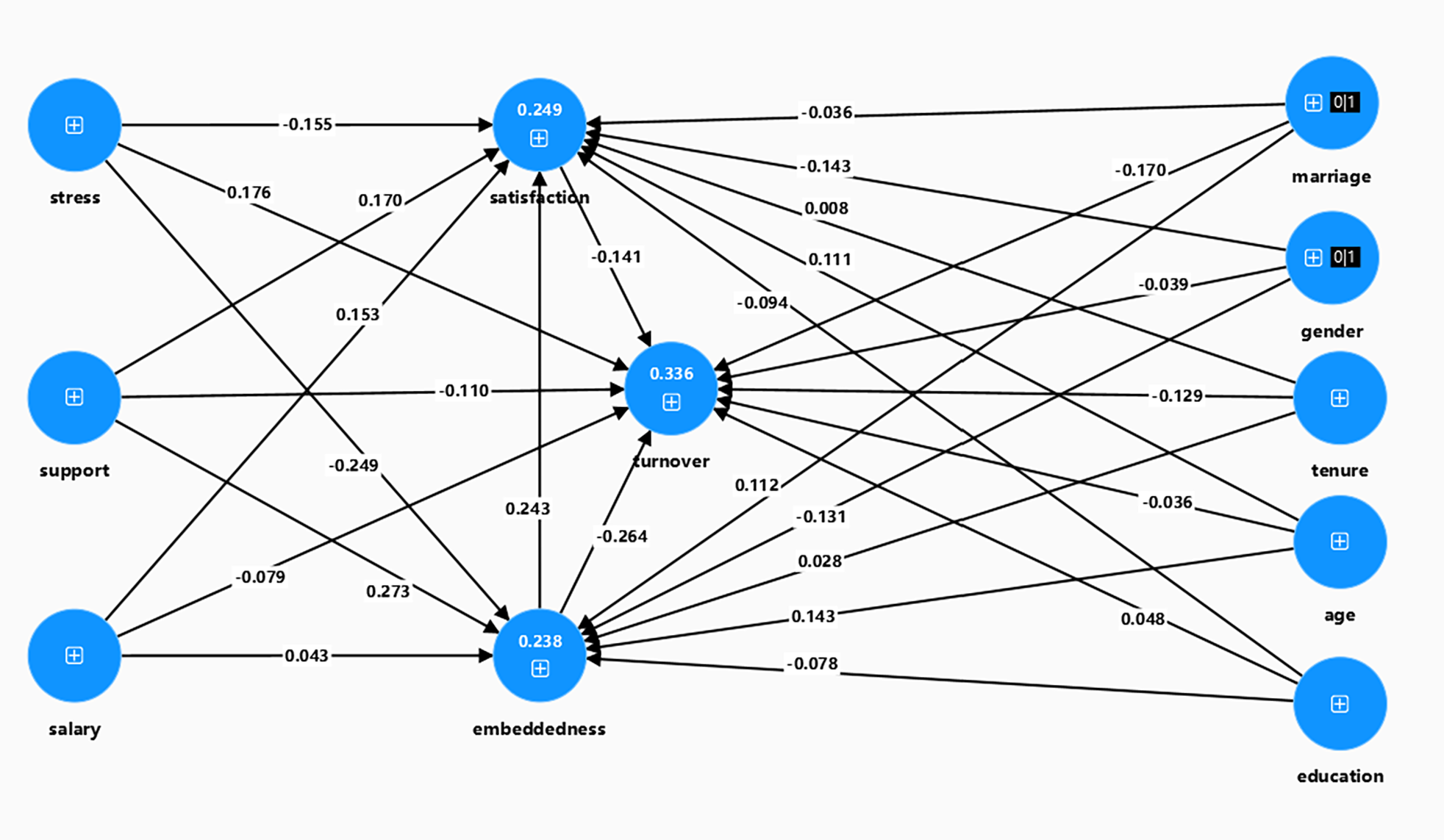


***Note.*** *Values on the arrows represent standardized path coefficients. Values inside the blue circles indicate the adjusted coefficient of determination (R^2^) for embeddedness, satisfaction, and turnover intention.*

**Supplementary Figure 2.** Direct effects of the proposed model (SmartPLS output). Values in parentheses represent the P-values of the path coefficients (95%CI).


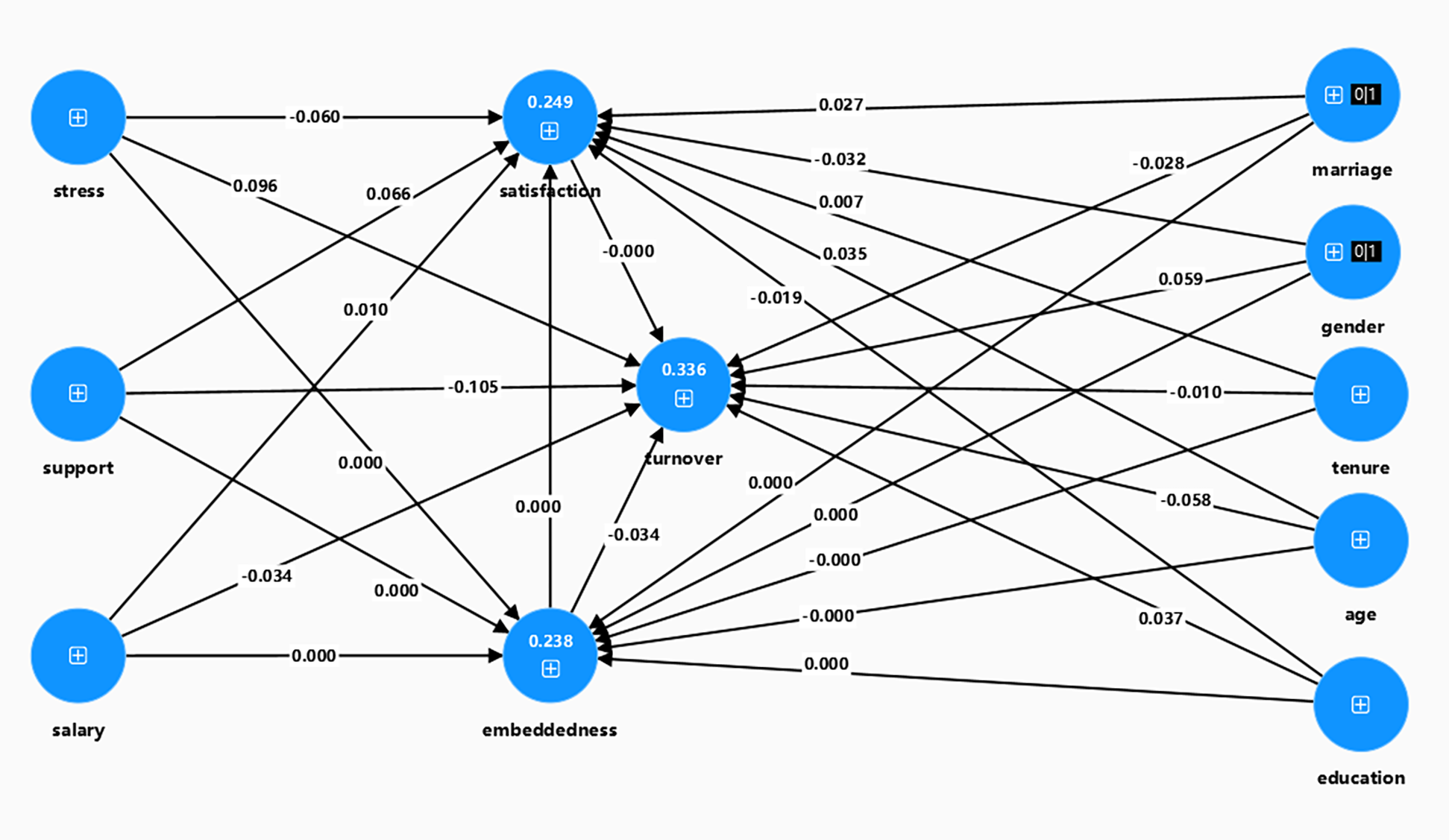


***Note.*** *Values on the arrows represent standardized path coefficients. Values inside the blue circles indicate the adjusted coefficient of determination (R^2^) for embeddedness, satisfaction, and turnover intention.*

**Supplementary Figure 3.** Indirect effects of the proposed model (SmartPLS output).
